# Analytical evaluation of two automatic methods to measure blood CK-MB mass and troponin I

**DOI:** 10.1155/S146392460200010X

**Published:** 2002

**Authors:** Amparo Galán, Antonio Curos, Jordi Duran, Augusto Corominas, Vicente Valle

**Affiliations:** 1 Department of Clinical Biochemistry Hospital Universitario Germans Trias i Pujol Badalona Barcelona Spain; 2 Coronary Care Unit Hospital Universitario Germans Trias i Pujol Badalona Barcelona Spain; 3 Servicio de Bioquímica Clínica Hospital Universitario Germans Trias i Pujol Carretera del Canyet s/n Badalona Barcelona Spain

## Abstract

The lack of standardization of methods to measure cardiac markers for coronaria ischaemia, particularly troponin, led us to perform an analytical evaluation of two new immunoassays to quantify CK-MB mass and troponin I using the Dimension R×L automatic analyser. The reliability and analytical intervals of the methods were studied as well as reference values (0.010— 0.228 μg l^-1^ for troponin I, 0.20—3.90 mg μg l^-1^ for CK-MB mass) and cuto¡ values (0.77 μg l^-1^ for troponin I, 5 μg l^-1^ for CK-MB mass) established. The cutoff values were established from 37 patients with acute myocardial infarction and from 20 with unstable angina. The absence of method cross-reactivity was corroborated using myocardial, brain and skeletal muscle tissue. Both methods were highly specific and showed good reliability and practicability in the diagnosis of coronaria ischaemia after 6 h of precordial pain.


Journal of Automated Methods & Management in Chemistry
Vol. 24, No. 2 (March–April 2002) pp. 51–57

Taylor & Francis

Taylor & Francis Group

### Analytical evaluation of two automatic methods to measure blood CK-MB mass and troponin I

**Amparo Galán[1]*, Antonio Curos[2], Jordi Duran[1], Augusto Corominas[1] and Vicente Valle[2]**
*[1]Department of Clinical Biochemistry and [2]Coronary Care Unit, Hospital Universitario Germans Trias i Pujol, Badalona, Barcelona, Spain*

*The lack of standardization of methods to measure cardiac markers for coronaria ischaemia, particularly troponin, led us to perform an analytical evaluation of two new immunoassays to quantify CK-MB mass and troponin I using the Dimension RxL automatic analyser. The reliability and analytical intervals of the methods were studied as well as reference values (0.010–0.228 µg l⁻¹ for troponin I, 0.20–3.90 µg l⁻¹ for CK-MB mass) and cutoff values (0.77 µg l⁻¹ for troponin I, 5 µg l⁻¹ for CK-MB mass) established. The cutoff values were established from 37 patients with acute myocardial infarction and from 20 with unstable angina. The absence of method cross-reactivity was corroborated using myocardial, brain and skeletal muscle tissue. Both methods were highly specific and showed good reliability and practicability in the diagnosis of coronaria ischaemia after 6 h of precordial pain.*

### Introduction

Only 10% of patients presenting in the emergency department with maintained precordial pain show an elevation in the ST segment of an electrocardiogram. In acute coronary ischaemia processes other than infarction with an elevated ST segment (non-Q infarction or without a fall in the ST, unstable angina and unidentified ischaemia), the use of biochemical markers may be the only criteria to diagnose myocardial necrosis and in some cases to establish the prognosis.

For many years, the enzymatic profile (creatine kinase, CK-MB isoenzyme activity, aspartate aminotransferase, lactate dehydrogenase) has been the biochemical method of choice i the diagnosis of acute myocardial infarction (AMI). Indeed, the World Health Organization (WHO) continues to use the catalytic CK-MB activity and creatine kinase in the diagnosis of AMI [1].

In the 1990s, more sensitive and specific cardiac markers have been developed to detect this diseases. A coronary ischaemia marker must fulfil the following characteristics: (1) a simple methodology providing rapid results 24 h a day; (2) a high diagnostic sensitivity, specially during the first 24 h of the ischaemic process; and (3) diagnostic specificity as close to 100% as possible. The following markers were used: serum myoglobin, CK-MB isoforms, CK-MB protein concentration (CK-MB mass), and troponins I and T. Each marker has pros and cons with respect to others and there is no consensus about the ideal marker. Myoglobin is the earliest marker and is highly sensitive in the diagnosis of the disease within 2 h of precordial pain [2, 3]. However, its diagnostic specificity is poor. The CK-MB isoforms are very early markers with good sensitivity within 4–6 h of precordial pain with relatively high specificity [4]. However, the technology used to perform this method is complicated [5] and expensive [6]. Protein concentration of CK-MB has markedly improved the analytical specificity and sensitivity of the activity of CK-MB methods [7, 8]. CK-MB mass has a high diagnostic sensitivity 4–6 h after precordial pain and remains increased for the first 48 h. The cardiospecificity of troponin [9] together with its wide diagnostic window (elevations persist in plasma from 6–12 h to 5–10 days for troponin I and from 6–12 h to 5–15 days for troponin T) have revolutionized the diagnosis of coronary ischaemia in the last few years [10–13].

In the 2000s, the Joint Committee of the European Society of Cardiology and the American College of Cardiology established redefined criteria to classify acute coronary syndromes [14] and they proposed troponin and CK-MB mass as biochemical markers of myocardial necrosis. Recently, Apple and Wu [15] stated that cardiac troponin is the best marker for diagnosis, risk stratification and guidance of therapy in acute coronary syndromes.

The correct choice of a cardiac marker according to the time of evolution of the ischaemic process is, currently, controversial, and the scientific societies are attempting to protocolize this choice [16–18]. The lack of method standarization [19, 20], specially measuring troponin, has made it difficult to compare the results and choose the best method [21]. In fact, scientific societies are, at present, elaborating primary [22] and reference materials [23] for cardiac markers, with the aim of achieving the transferability of results and establishing the diagnostic limits to facilitate the clinical use of these markers. The National Academy of Clinical Biochemistry (NACB) has suggested that two cutoff values for the troponins be used, one indicative of AMI and the other indicative of cardiac injury [17]. Accurate discrimination between minor myocardial injury versus analytical imprecision requires high interassay precision at the low cutoff values. Thus, the NACB has recommended a precision for cardiac troponin assays of $\leq 10\%$ [17].

Therefore, each laboratory should evaluate its methods, especially with troponin I, and establish its reference ranges and cutoff values as well as the clinical sensitivity

*To whom correspondence should be addressed at: Servicio de Bioquímica Clínica, Hospital Universitario Germans Trias i Pujol, Carretera del Canyet s/n, Badalona, Barcelona, Spain. e-mail: agalan@ns.hugtip.scs.es

*Journal of Automated Methods & Management in Chemistry* ISSN 1463–9246 print/ISSN 1464–5068 online © 2002 Taylor & Francis Ltd
http://www.tandf.co.uk/journals
DOI: 10.1080/14639240210121833

and specificity for the different processes of acute coronary ischaemia [21]. In the present study, we performed an analytical evaluation of two new methods to measure the protein concentration of CK-MB mass and cardiac troponin I using the Dimension RxL automatic analyser (Dade Behring, Newark, DE, USA).

### Materials and methods

### *Patients*

We investigated 37 patients with confirmed AMI admitted to the coronary care unit (CCU). The definitive diagnosis of AMI required, at least, two of the following three clinical criteria proposed by the WHO [1]: typical prolonged severe chest pain of > 20-min duration, the evolution of abnormal Q waves or equivalents on serial ECGs, and serial CK and CK-MB elevations with an initial rise and subsequent fall peak. Twenty patients with a clinical diagnosis compatible with unstable angina were included in the study. Blood was withdrawn at different time intervals after precordal pain.

Reference values were calculated from 50 samples obtained from apparently normal people free of cardiological diseases sent to our laboratory for analytical control before minor surgical interventions.

To study the cross-reactivity of the methods, samples of myocardial, brain and skeletal muscle (biceps, quadriceps) from a subject were obtained at autopsy 20 h after death. The patient had died in an accident. Ethical permission for tissue donation was previously obtained. Samples were frozen at –80 °C until their analyses.

### *Analytical methods*

- Troponin I (cat. no. RF421; Dade Behring). The immunoassay was based on the sandwich principle with two monoclonal antibodies binding to a conjugate reagent composed with alkaline phosphatase. The coloured final product absorbed at 510 nm.
- Protein concentration of CK-MB (CK-MB mass) (cat. no. RF420; Dade Behring). The immunoassay was based on the sandwich principle with a monoclonal antibody specific for MB isoenzyme and another against the B subunit of creatine kinase. The detection system is β-galactosidase.
- Catalitic activity of CK-MB (cat. no. DF31; Dade Behring). The immunoinhibition method detected the B subunit of creatine kinase.
- Creatine kinase activity (CK) (cat. no. DF29A; Dade Behring).

The assays were carried out in an automatic analyser Dimension RxL.

### *Analytical evaluation of the methods*

The analytical intervals of the methods were evaluated following the directions of the Socité Française de Biologie Clinique [24]. To calculate the analytical interval, nine standard aqueous solutions of troponin I (cardiac troponin I calibrator; Dade Behring cat. no. RC421B) ranging from 0.1 to 60 $\mu g\,l^{-1}$ and of CK-MB (mass creatine kinase MB isoenzyme calibrator Dade Behring cat. no. RC420) (1–400 $\mu g\,l^{-1}$) were used. The recommendations of the European Committee for Clinical Laboratory Standards (ECCLS) were followed for the study of the imprecision and inaccuracy of the method [25]. Control serums of 0.34, 0.68, 2.42, 9.7 and 22.6 $\mu g\,l^{-1}$ troponin I and 2, 4.9, 7.5, 15.1 and 42.6 $\mu g\,l^{-1}$ CK-MB mass were used.

To determine the analytical recovery of the methods, increasing the quantities of troponin I to CK-MB mass were added to different aliquots of sera pool. A total of 20, 30 and 40 $\mu g\,l^{-1}$ troponin I and 50, 100 and 200 $\mu g\,l^{-1}$ CK-MB mass were the standard aqueous solutions used. The dilution effect was corrected by adding the same volume of saline solution to unaltered samples.

The reference values of the methods were obtained following the recommendations of the Panel of Experts of the IFCC on reference values [26].

### *Tissue specificity of the methods*

Cross-reactivity for CK-MB mass method was carried out with homogenates from myocardial, brain and skeletal muscle tissue. The crude extracts preparation was performed following the previous protocol used for myocardial tissue [27, 28]. Tissue was homogenized in 2 vols 50 $mmol\,l^{-1}$ Tris-HCl buffer, pH 8.0, containing 2 $mmol\,l^{-1}$ 2-mercaptoethanol with a Polytron ATA 20 TSM homogenizer (Kinematica Gmb, Littau, Switzerland). Homogenization was carried out in an ice bath at 4 °C for 45 min. After centrifugation of the homogenate at 4000*g* for 15 min in a Beckman J2-21 centrifuge (Beckman Instruments, Palo Alto, CA, USA), the supernatant was collected and filtered. The protein concentration of CK-MB and the catalytical activity of total creatine kinase were measured in the supernatant.

The cardiospecificity of troponin was verified using myocardial and skeletal muscle (quadriceps, biceps) tissue. Troponin I was measured in myofibrillar and cytosolic fractions of these tissues. To separate these fractions, the protocol of Katus *et al.* was applied [29]. The tissue was homogenized in a buffer (50 $mmol\,l^{-1}$ Tris-HCl, 2 $mmol\,l^{-1}$ EDTA, 0.05 $mmol\,l^{-1}$ dithiotreitol, pH 7.0) and stirred at 4 °C for 1 h. The insoluble molecules were precipitated by ultracentrifugation for 1 h at 100 000*g* (4 °C). The pellet was washed and centrifugation was repeated. The troponins contained in the myofibrillar fraction were extracted by homogenization of the precipitate for 1 h at 4 °C in a solution composed by 0.4 $mol\,l^{-1}$ potassium chloride, 0.01 $mol\,l^{-1}$ magnesium chloride, 0.1 $mol\,l^{-1}$ potassium dihydrogen phosphate, 0.05 $mol\,l^{-1}$ potassium monohydrogen phosphate, 0.04 $mol\,l^{-1}$ sodium pyrophosphate, pH 7.0. The solubilized troponin complex was separated from actomyosin by centrifugation for 1 h at 20 000*g* at 4 °C. Troponin I concentration was measured in the myofibrillar and soluble fractions as well as in the myocardial and skeletal muscle homogenates. The protein content was measured by a pirogallol red method [30].

*Diagnostic decision limits*

The decision limits for the markers were obtained using receiver operating characteristic (ROC) curves.

*Statistical methods*

Mean, SD and coefficient of variation for studying the accuracy and imprecision were used. Analytical intervals were determined by regression analyses. The Mann–Whitney U-test was applied to establish the statistical differences between groups. To calculate the reference values of the method, the 2.5, 50 and 97.5 percentiles were used. ROC curves were applied to obtain the cutoff values. Confidence intervals (95%) of sensitivities were calculated non-parametrically. The $\chi^2$-test was used to compare the percentages of sensitivity.

### Results

*Analytical interval of methods*

The analytical interval for CK-MB mass ranged from 0.1 to 341 μg l$^{-1}$. Figure 1 shows the linear regression of the least-squares ($y = 0.978x + 0.24$; $r = 0.999$) established among the theoretical values of the CK-MB standards processed in triplicate over 3 consecutive days, and the values found. The analytical interval for troponin I was 0.05–50 μg l$^{-1}$ (figure 2).

*Imprecision and inaccuracy*

Table 1 contains the variation coefficients for the troponin I method. The study of imprecision for the CK-MB method is summarized in table 2. The percentages of inaccuracy with respect to the theoretical value did not exceed 10% (tables 3 and 4).

*Analytical recovery study.* Analytical recovery ranged from 98.7 to 102% for troponin I (table 5) and 98–103% for CK-MB mass (table 6).

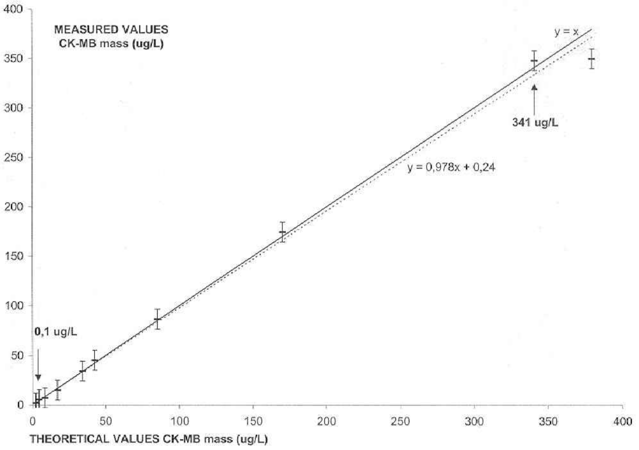

*Figure 1. Analytical interval for CK-MB mass. Reference line (−) y = bx (b = 1). Regression line (.....) mean and 2 SD of each point (┼). The means of the points found and their 2 SD coincide with the reference line from 0.1 to 341 μg l$^{-1}$*

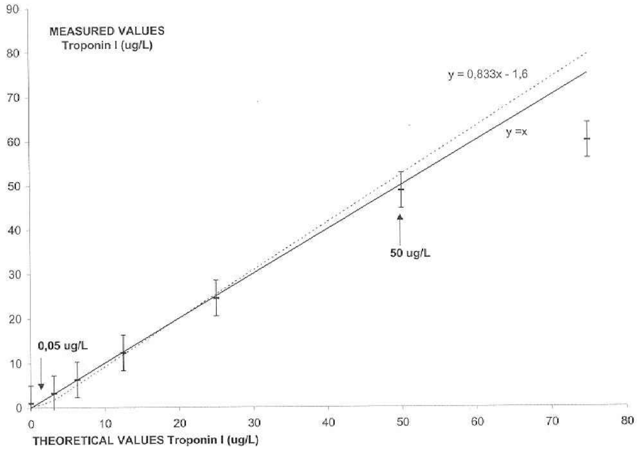

*Figure 2. Analytical interval for troponin I. Reference line (−) y = bx (b = 1). Regression line (.....) mean and 2 SD of each point (┼). The means of the points found and their 2 SD coincide with the reference line from 0.05 to 50 μg l$^{-1}$.*

*Table 1. Imprecision of the troponin I method.*

| Concentration of troponin I (μg l$^{-1}$) | *n* | Within-run CV (%) | Between-run CV (%) |
|---|---|---|---|
| 0.34 | 20 | 9.8 | 11.2 |
| 0.68 | 20 | 5.0 | 5.5 |
| 2.42 | 20 | 4.3 | 4.9 |
| 9.7 | 20 | 3.7 | 4.7 |
| 22.6 | 20 | 4.8 | 4.9 |

CV, coefficient of variation.

*Table 2. Imprecision of the CK-MB mass method.*

| Concentration of CK-MB mass (μg l$^{-1}$) | *n* | Within-run CV (%) | Between-run CV (%) |
|---|---|---|---|
| 2.0 | 20 | 9.5 | 11.8 |
| 4.0 | 20 | 4.9 | 5.0 |
| 7.5 | 20 | 3.1 | 4.0 |
| 15.1 | 20 | 4.5 | 6.7 |
| 42.6 | 20 | 4.7 | 4.8 |

CV, coefficient of variation.

*Table 3. Inaccuracy of the troponin I method.*

| Theoretical concentration of troponin I (μg l$^{-1}$) | *n* | Found concentration of troponin I (μg l$^{-1}$) | Inaccuracy (%) |
|---|---|---|---|
| 0.34 | 20 | 0.325 | −4.4 |
| 9.7 | 20 | 10.15 | 4.6 |
| 22.6 | 20 | 23.2 | 2.6 |

*Table 4. Inaccuracy of the CK-MB mass method.*

| Theoretical concentration of CK-MB mass ($\mu g\, l^{-1}$) | $n$ | Found concentration of CK-MB mass ($\mu g\, l^{-1}$) | Inaccuracy (%) |
|---|---|---|---|
| 4.0 | 20 | 4.19 | 4.75 |
| 15.1 | 20 | 14.4 | −4.6 |
| 42.6 | 20 | 43.1 | 1.1 |

*Reference values*

The reference values for the cardiac markers studied are contained in table 7. Statistical differences between men and women groups were not found for any marker: troponin I ($p > 0.05$), CK-MB mass ($p > 0.05$), CK-MB mass/total CK ($p > 0.05$). Thus, the reference values were established in the total population.

*Crossreactivity*

The CK-MB mass method showed the absence of cross-reactivity with CK-BB and CK-MM. CK-MB concentration in myocardial extract was similar before and after the addition of 1500 U $l^{-1}$ CK-BB from brain tissue or to 12 000 U $l^{-1}$ CK-MM from skeletal muscle tissue (figure 3). The absence of troponin I in skeletal muscle was corroborated: the concentration of troponin I in biceps and quadriceps was not detected (< 1% of the values obtained in myocardial tissue). The troponin I concentration in the myofibrillar fraction of cardiac tissue was 7.2 mg $g^{-1}$ protein and only 4% of total troponin content was found in the soluble fraction (figure 4).

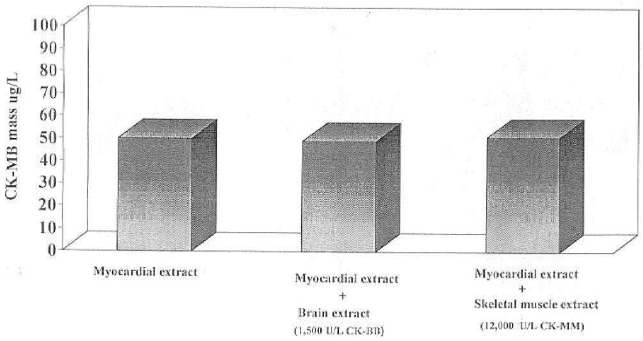

*Figure 3. Tissue specificity for the CK-MB mass method. No differences were found between the CK-MB mass concentration of the crude extract of myocardial tissue before and after adding 1500 U $l^{-1}$ of CK-BB ($t = 0.12$, $p = 0.885$) and 12 000 U $l^{-1}$ of CK-MM ($t = 0.21$, $p = 0.771$).*

*Table 5. Recovery study for troponin I.*

| Serum pool ($\mu mol\, l^{-1}$) | Troponin I added ($\mu mol\, l^{-1}$) | Theoretical troponin I concentration ($\mu mol\ l^{-1}$) | Troponin I concentration found ($\mu mol\ l^{-1}$) | Recovery (%) |
|---|---|---|---|---|
| 50 | 20 | 70 | 71 | 101 |
| 50 | 30 | 80 | 79 | 98.7 |
| 50 | 40 | 90 | 92 | 102 |

*Table 6. Recovery study for CK-MB mass.*

| Serum pool ($\mu mol\, l^{-1}$) | CK-MB mass added ($\mu mol\, l^{-1}$) | Theoretical CK-MB mass concentration ($\mu mol\, l^{-1}$) | CK-MB mass concentration found ($\mu mol\, l^{-1}$) | Recovery (%) |
|---|---|---|---|---|
| 80 | 50 | 130 | 134 | 103 |
| 80 | 100 | 180 | 182 | 101 |
| 80 | 200 | 280 | 276 | 98 |

*Table 7. Reference values.*

| | Males ($n = 42$) | Females ($n = 48$) | $p$ | Total ($n = 90$) |
|---|---|---|---|---|
| Troponin I ($\mu g\, l^{-1}$) | | | | |
| Range (p2.5, p97.5) | 0.011–0.227 | 0.010–0.225 | >0.05 | 0.010–0.228 |
| Mean (p50) | 0.052 | 0.053 | | 0.051 |
| CK-MB mass ($\mu g\, l^{-1}$) | | | | |
| Range (p2.5, p97.5) | 0.21–3.5 | 0.19–4.0 | >0.05 | 0.20–3.90 |
| Mean (p50) | 0.911 | 0.890 | | 0.900 |
| CK-MB mass/CK (%) | | | | |
| Range (p2.5, p97.5) | 0.38–4.6 | 0.43–4.9 | >0.05 | 0.42–4.82 |
| Mean (p50) | 1.5901 | 1.6002 | | 4.6061 |

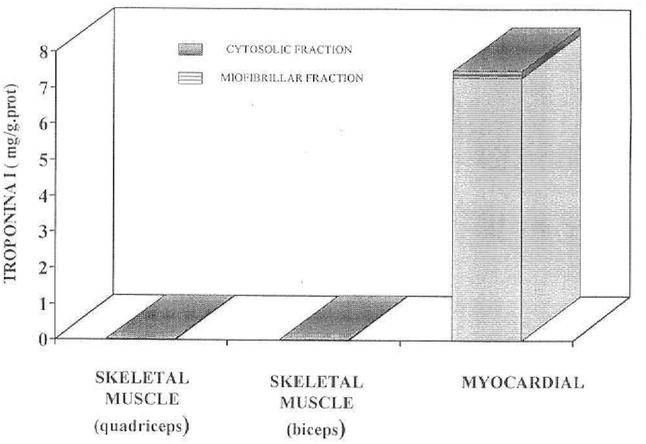

*Figure 4. Tissue specificity for the troponin I method. The lack of cross-reactivity of troponin I with skeletal muscle is shown. No troponin I concentrations in the crude extract of biceps and quadriceps were detected. The troponin I concentration in the myofibrillar fraction of cardiac tissue was 7.2 mg g$^{-1}$ protein and in the cytosolic fraction it was 0.3 mg g$^{-1}$ protein.*

*Diagnostic decision limits*

ROC curves were applied to establish the diagnostic decision limits of the methods. Table 8 shows the percentages of sensitivity of troponin I for cardiac markers in AMI using the cutoff values that established (0.228 and 0.77 μg l$^{-1}$) and the cutoff value proposed by the manufacturer (1.5 μg l$^{-1}$). In the early 6 h of precordial pain, the sensitivity of troponin I, using the reference value (0.228 μg l$^{-1}$) as a cutoff, was similar to CK-MB mass ($\chi^2 = 0.82$, $p > 0.05$). However, the sensitivities of troponin I using 0.77 and 1.5 μg l$^{-1}$ were lower and significantly different than CK-MB mass ($\chi^2 = 12$, $p < 0.01$; and $\chi^2 = 34$, $p < 0.001$, respectively). After 7 h of precordial pain, the sensitivity was reliable using any cutoff value.

Table 9 contains the percentage of patients with unstable angina and the increase of cardiac marker values. No differences were found ($p > 0.05$) between the percentages of sensitivity for troponin I using 0.228, 0.77 or 1.5 μg l$^{-1}$ as the cutoff. The sensitivity for CK-MB mass was lower than troponin I ($p < 0.05$) to diagnose minimum myocardial damage.

### Discussion

The CK-MB mass method presented demonstrates good analytical precision for concentrations with clinical pathological significance of the isoenzyme. However, the variation coefficient within the normal limits is > 10%, similar to that obtained by other authors for other CK-MB mass methods [31]. At levels that were slightly higher than our reference 0.34 μg l$^{-1}$, we found analogically variation coefficients of 11.2% for the troponin I method. Nonetheless, > 0.68 μg l$^{-1}$, the imprecision of the assay is < 5% with the variation coefficient obtained being better than that reported for other commercially available troponin tests [16, 21]. More-

*Table 8. Sensitivites (95% confidence intervals) of biochemical markers in AMI at different hours after precordial pain and using different cutoff values.*

| Time interval (h) | *n* | Troponin I (cutoff value 0.228 μg l$^{-1}$) (%) | Troponin I (cutoff value 0.77 μg l$^{-1}$) (%) | Troponin I (cutoff value 1.5 μg l$^{-1}$) (%) | CK-MB mass (cutoff value 5 μg l$^{-1}$) (%) |
|---|---|---|---|---|---|
| 2–6 | 27 | 88 (75–97) | 44.4 (27–61) | 22.2 (14–48) | 90 (77–89) |
| 7–11 | 37 | 100 (91–100) | 100 (91–100) | 95 (82–99) | 95.1 (80–99) |
| 12–15 | 27 | 100 (91–100) | 100 (91–100) | 100 (91–100) | 100 (91–100) |
| 16–20 | 37 | 100 (91–100) | 100 (91–100) | 100 (91–100) | 100 (91–100) |
| 21–24 | 27 | 100 (91–100) | 100 (91–100) | 100 (91–100) | 100 (91–100) |

*Table 9. Increase of cardiac markers in patients with unstable angina. Percentage of sensitivity and 95% confidence intervals.*

| Time interval after precordial pain (h) | *n* | Troponin I (cutoff value 0.228 μg l$^{-1}$) (%) | Troponin I (cutoff value 0.77 μg l$^{-1}$) (%) | Troponin I (cutoff value 1.5 μg l$^{-1}$) (%) | CK-MB mass (cutoff value 5 μg l$^{-1}$ l) (%) |
|---|---|---|---|---|---|
| 6 +/− 3 | 20 | 33 (20–49) | 33 (20–49) | 26 (10–38) | 3 (0–9) |
| 14 +/− 3 | 20 | 55 (37–71) | 55 (37–71) | 31 (9–40) | 5 (1–10) |
| 22 +/− 3 | 20 | 50 (32–66) | 50 (32–66) | 33 (18–50) | 5 (0.5–11) |

over, the response time for the measurement of these markers is < 20 min, fulfilling the norms proposed by the International Society of Enzymology [32] for cardiac marker response time and is therefore compatible with the results required in emergency department laboratories [17].

On the study of CK-MB cross-reactivity, we found that contrary to methods analysing the catalytic activity of CK-MB, the monoclonal antibody used in our CK-MB mass method exclusively recognized the MB isoenzyme and not the BB and MM isoenzymes of creatine kinase. This method, therefore, improves the metrologic specificity of the assay with regard to activity, although the CK-MB mass is not 100% cardiospecific. The skeletal muscle contains small, but significant, quantities (1–3%) of the isoenzyme [33] and, thus, following massive musculoskeletal lesions, this marker is elevated [34]. We have also shown that the troponin I method evaluated herein lacks cross-reactivity with human skeletal muscle (biceps, quadriceps).

As a myofibrillar protein, troponin I may be present in different forms in blood (proteolytic fragments, intact molecules, phosphorylated proteins or forming complexes with other myofibrillar proteins). To do so, different recoveries of the respective antigen are obtained according to the monoclonal antibody used. The currently available commercial kits to measure troponin I differ in the antigenic specificities of the selected antibodies and in the recovery of the fragments and complexes of troponin I. This indicates the need for standardization of the assays determining this marker and in the meantime each laboratory must establish their own reference values and diagnostic decisions limits. For the method evaluated, we have established reference values of between 0 and 0.28 $\mu g\,l^{-1}$, which are higher than those proposed by the manufacturer (0–0.05 $\mu g\,l^{-1}$). The differences between the reference value of the different assays in the market may range to up to 10-fold [12, 21, 35, 36]. The choice of a cutoff point is one of the most widely debated subjects [17, 37–40]. From our results, it may be seen that the cutoff point of 1.5 $\mu g\,l^{-1}$ proposed by the manufacturer of the assay evaluated provides a diagnostic sensitivity of only 22% for AMI during the first 6 h of the ischaemic process. Moreover, the detection of the small myocardial lesions with this cutoff point is lower than that found when using the diagnostic cutoff values we have established, although the difference is not significant. The best sensitivity for the diagnosis of AMI is that obtained with 0.228 $\mu g\,l^{-1}$ (88% in the first 6 h of the ischaemic process). However, the imprecision of the method is > 10% at this concentration. Therefore, although sensitivity is lost at 0.77 $\mu g\,l^{-1}$, since it is of 44.4% in the first 6 h of the pain, the reliability of the method is much greater at these levels. Moreover, according to our results, the sensitivity of the marker for the small myocardial lesions is similar if either 0.228 or 0.77 $\mu g\,l^{-1}$ are used as the diagnostic cutoff value. Nonetheless, our results agree with the criteria of many authors [17, 18, 41] in that to obtain the true diagnostic value of troponin I, > 6 h of the ischaemic process must go by. In fact, it is after 7 h that our results show a diagnostic sensitivity of 100% for AMI. The late appearance of troponin in blood may be due to its profound cellular localization since the 94% of troponin T and the 97% of troponin I are largely bound to the tropomyosin complex and only the remaining 6 and 3%, respectively, are found in the cytoplasm. In our study with cardiac tissue, we only detected 4% of troponin I in the soluble fraction in tissue. The wide diagnostic window of troponin (10–15 days) may be due to its profound localization. The cutoff value of 5 $\mu g\,l^{-1}$ for CK-MB mass, a method which is more standardized than troponin, agrees with that proposed by the manufacturer and some authors working with other commercial kits [42, 43]. We have demonstrated that the sensitivity of CK-MB mass for the diagnosis of AMI during the first 6 h of precordial pain is higher than troponin I using 0.77 and 1.5 $\mu g\,l^{-1}$ as the cutoff values. Other authors [42, 44–46] have had similar results using other commercial kits. It must be taken into account that although CK-MB has a greater molecular mass than troponin, its intracellular localization is cytoplasmatic, thus favouring its release to the circulation. In contrast, the possible localization of CK-MB in skeletal muscle decreases the specificity of the test for the diagnosis of coronary ischaemia.

In conclusion, the methods studied are highly specific, and show good reliability and practicability for the emergency diagnosis of acute coronary ischaemia. With 120 (l, sample results of the two cardiac markers may be obtain in 16 min. Both markers showed a reliable diagnostic sensitivity for acute myocardial infarction after 6 h of precordial pain. However, we proposed using 0.77 instead off 1.5 $\mu g\,l^{-1}$ as the cutoff value for troponin I.

### References

1. Report of the Joint International Society and Federation of Cardiology/World Health Organization Task Force on Standardization of Clinical Nomenclature. *Circulation*, **59** (1979), 607.
2. Mair, J., *Crit. Rev. Clin. Lab. Sci.*, **34** (1997), 66.
3. Montague, C. and Kircher, T., *Am. J. Clin. Path.*, **104** (1995), 472.
4. Wu, A. H. B., Wang, X. M., Gornet, T. G. and Ordoñez-Llanos, J., *Clin. Chem.*, **38** (1992), 2396.
5. Panteghini, M., *Clin Chem*, **33** (1988), 1284.
6. Escobar, R., Gornet, T. G. and Wu, A. H. B., *Clin. Chem.*, **33** (1988), 1284.
7. Henderson, A. R., Krishnan, S., Webb, S., Cheung, C. M., Nazir, D. J. and Richardson, H., *Clin. Chem.*, **44** (1998), 124.
8. Galán, A., Balsells, R., Ferragut, F. J., Gella, F. J., Gubern, G., Padrós, A. and Rueda, R., *Quim. Clin.*, **15** (1996), 53.
9. Wu, A. H. B., *Clin. Biochem.*, **30** (1997), 339.
10. Ohman, E. M., Armstrong, P. W., Christenson, R. H., Granger, C. B., Katus, H. A., Hamm, A. W., O'Hanesian, M. A., Wagner, G. S., Kleiman, N. S., Harrell, F. E, Califf, R. M. and Topol, E. J., *N. Engl. J. Med.*, **335** (1996), 1342.
11. Antman, E. M., Tanasijevic, M. J., Thompson, B., Schactman, M., McCabe, C. H., Cannon, C. P., Fischer, G. A., Iung, A. Y., Thompson, B. S. C., Wybenga, D. and Braunwald, E., *N. Engl. J. Med.*, **335** (1996), 1333.
12. Henderson, A. R., *Clin. Lab. Med.*, **17** (1997), 625.
13. Hamm, C. W., in E. Braunwald (ed.), *Heart Disease: a Textbook of Cardiovascular Medicine*, 5th edn (Philadelphia, PA: W. B. Saunders, 1997), update 3,1.
14. The Joint European Society of Cardiology/American College of Cardiology Committee, *Euro. Heart. J.*, **21** (2000), 1502.
15. Apple, F. and Wu, A. H. B., *Clin. Chem.*, **47** (2001), 377.
16. Panteghini, M., *Clin. Chem. Lab. Med.*, **36** (1998), 887.

17. Wu, A. H. B., Apple, F. S., Gibler, W. B., Jesse, R. L., Warshaw, M. M. and Valdes, R., *Clin. Chem.*, **45** (1999), 1104.
18. Panteghini, M., Apple, F. S., Christenson, R. H., Dati, F., Mair, J. and Wu, A. H., *Clin. Chem. Lab. Med.*, **37** (1999), 687.
19. Panteghini, M., *Clin. Chim. Acta.*, **311** (2001), 19.
20. Wu, A. H. B., in *Markers in Cardiology: Current and Future Clinical Applications* (Armonk, NY, 2001), 1, 1.
21. Apple, F. S., Adams, J. E., Wu, A. H. B. and Jaffe, A. S., in *Markers in Cardiology: Current and Future Clinical Applications* (Armonk, NY, 2001), 4, 31.
22. Dati, F., Panteghini, M., Apple, F. S., Christenson, R. H., Mair, J. and Wu, A. H., *Scand. J. Clin. Lab. Invest.*, **59** (suppl. 230) (1999), 113.
23. Christenson, R. H., Duh, S. H., Apple, F. S., Bodor, G. S., Bunk, D. M., Dalluge, J., Panteghini, M., Potter, J. D., Welsh, M. J., Wu, A. M. and Kahn, S. E., *Clin. Chem.*, **47** (2001), 431.
24. 17cc Socité Franaise de Biologie Clinique.Commission, *Ann. Biol. Clin.*, **44** (1986), 686.
25. 18cc European Committee for Clinical Laboratory Standards. *Guidelines for the Evaluation of Analyzers in Clinical Chemistry.* ECCLS Document 3, 1986, 2.
26. 19cc International Federation of Clinical Chemistry, *Clin. Chem. Acta.*, **170** (1987), S13.
27. Galán, A., Canalias, F., Padros, A. and Sierra, C., *Rev. Diag. Biol.*, **3** (1996), 159.
28. Gella, F. J., Frey, E., Ceriotti, F., Galán, A., Hadjivassiliou, A. G., Horder, M., Lorentz, K., Moss, D. N., Schiele, F. and Canalias, F., *Clin. Chim. Acta.*, **276** (1998), 35.
29. Katus, H. A., Remppis, A., Scheffold, T., Diederich, K. W. and Kuebler, W., *Am. J. Cadiol.*, **67** (1991), 1360.
30. Fujita, Y., Morl, I. and Kitano, S., *Bunseki Kagaku*, **32** (1983), 379.
31. Ross, S. M. and Fraser, C. G., *Ann. Clin. Biochem.*, **35** (1998), 80.
32. Henderson, A. R., Gerhardt, W. and Apple, F. S., *Clin. Chim. Acta.*, **272** (1998), 93.
33. Panteghini, M., *Curr. Opin. Rheumatol.*, **7** (1995), 469.
34. Adams, J. E. III, Bodor, G. S., Dávila-Román, V. G., Delmez, J. A., Apple, F. S., Ladenson, J. H., Ladenson, J. H. and Jaffe, A. S., *Circulation*, **88** (1993), 101.
35. Waskiewicz, D., Lavigne, L., Salhaney, J., Annunziato, M., Fagan, G. and Whiteley, G., *Clin. Chem.*, **41** (1995), S63.
36. Flaa, C., Sabucedo, A., Geist, W., DeMarco, C., Troy, S., Chavailaz, P. and Bauer, R., *Clin. Chem.*, **39** (1993), 2435.
37. Apple, F., *Clin. Chem.*, **45** (1999), 45.
38. Wu, A., Feng, Y., Moore, R., Apple, F., McPherson, P., Buechler, K. and Bodor, G., *Clin. Chem.*, **44** (1998), 1198.
39. Katrukha, A., Bereznikova, A. and Pettersson, K., *Scand. J. Clin. Lab. Invest.*, **59** (suppl. 230) (1999), 124.
40. Jaffe, A. S., in *Markers in Cardiology: Current and Future Clinical Applications* (Armonk, NY, 2001), 10,63.
41. Christenson, R. H. and Duh, S.-H., *Scand. J. Clin. Lab. Invest.*, **59** (suppl. 230) (1999), 90.
42. Tucker, J. F., Collins, R. A. and Anderson, A. J., *Acad. Emerg. Med.*, **4** (1997), 13.
43. Brandt, D. R., Gates, R. C., Eng, K. K., Forsythe, C. M., Korom, G. K., Nitro, A. S., Koffler, P. A. and Ogundo, E. A., *Clin. Chem.*, **36** (1990), 375.
44. Mair, J., Genser, N., Morandell, D., Maier, J., Mair, P., Lechleitner, P., Calzolari, C., Larue, C., Ambach, E., Dienstl, F., Pau, B. and Puschendorf, B., *Clin. Chim. Acta.*, **245** (1996), 19.
45. Mair, J., Morandell, D., Genser, N., Lechleitner, P., Dienstl, F. and Puschendorf, B., *Clin. Chem.*, **41** (1995), 1266.
46. Panteghini, M., *Clin. Chim. Acta.*, **272** (1998), 23.

